# Comparison of the Effect of Mindfulness-based Cognitive Therapy Accompanied by Pharmacotherapy With Pharmacotherapy Alone in Treating Dysthymic Patients

**DOI:** 10.5812/ircmj.8024

**Published:** 2013-03-05

**Authors:** Sajedeh Hamidian, Abdollah Omidi, Seyyed Masoud Mousavinasab, Ghasem Naziri

**Affiliations:** 1 Department of Psychiatry and Psychology, Shiraz University of Medical Sciences, Shiraz, IR Iran; 2 Department of Psychiatry, Kashan University of Medical Sciences, Kashan, IR Iran; 3 Sciences and Researches Branch, Islamic Azad University Fars, Shiraz, IR Iran

**Keywords:** Dysthymia Disorder, Cognitive Therapy, Attention

## Abstract

**Background:**

Dysthimia in adults is a chronic depression disorder which is characterized by a mild depression for at least 2 years. Remarkable psycho-social involvements, greater disturbances in psycho-social functions compared to other forms of depression and lack of definite findings about preferred treatment for this disorder led us to evaluate the effectiveness of Mindfulness based cognitive therapy (MBCT) method adjunct to pharmacotherapy compared with pharmachothrapy alone in treating dysthymia in this thesis.

**Objectives:**

This study aimed to evaluate the effectiveness of mindfulness-based cognitive therapy on a chronic type of depression disorder called dysthymia

**Patients and Methods:**

This study is a clinical trial of an interventional method which was carried out on dysthymic and double depressed patients who had referred to psychiatric clinics of Shiraz University of Medical Sciences, Shiraz, Iran. In doing so, 50 patients above the age of 18 were selected through convenience sampling and assigned into intervention and control groups. The control group only received medications while the intervention group in addition to receiving medication, participated in 8 sessions of a mindfulness based cognitive therapy course which was held once a week and each session lasted for 2 to 2.5 hours. All the participants filled out Beck Depression Inventory II and five facet mindfulness questionnaire. The data were analyzed using the SPSS statistical software (version 16) and univariate covariance and independent t test statistical methods.

**Results:**

In this study, no statistically significant differences were found between the two groups regarding the demographic characteristics. The mean difference between the two groups was statistically significant for the variables in post-test considering the pre-test. The experimental group participants showed significant improvement in terms of the defined variables; a trend which was not observed in the control group participants.

**Conclusion:**

The results of this study show that adding MBCT to pharmacotherapy in treatment of dysthymic patients can cause significant improvement in depression symptoms and mindfulness skills in patients compared to pharmacotherapy alone.

## 1. Background

Dysthymia is a chronic disorder which is identified by a depressed mood which lasts most of the time of the day. It is a prevalent disorder with a 3% annual and 6% life time prevalence. 36% of psychiatric outpatients suffer from depression. In comparison to major depression, chronic depression has a longer treatment period and is accompanied by poor physical health, greater and severe disturbances in social, emotional, and psychological performances (1). Studies have shown that the indicators of dysthymia are mostly cognitive-emotional ones rather than motor and vegetative signs which can be observed in major depression (2). Moreover, disturbed quality of life, high levels of inability, insufficient social support and weak clinical as well as marital compatibility are considered as the psychological adverse effects of dysthymia (3). Dysthymia is different from melancholy which is identified by severe disorders in psychological, motor and vegetative functions. This is in fact a familiar dichotomy between endogenous and neurotic depression. In general, endogenous depression has a periodical nature as well as complete symptoms, while neurotic depression shows a weaker and more variable period (4). Up to 1980, the second group was considered as melancholy due to the chronic nature of the disease and was called “existential depression” (5). Most dysthymic patients experience at least one cycle of major depression (5-9) which is called double depression (5, 10). Such patients' disease pattern includes several years of dysthymia followed by one or more cycles of major depression. Of course, major depression may be recurrent and among the major depression cycles, dysthymia may return in the patients (6, 11). In general, insidious beginning of depression goes back to the late childhood or adolescence and most dysthymic patients complain about being depressed since birth. As Kurt Schneider mentions, these patients believe themselves to belong to an “aristocracy of suffering” and, in the absence of major depression symptoms, such descriptions of chronic gloominess result in these patients' being labeled with character logical depression (12). Considering treatment, dysthymia is considered to be a chronic treatment-resistant disorder and 40% of the patients do not respond to medication (13). Studies conducted on the issue also confirm that treatment of dysthymia is far more difficult than major depression (14). Moreover, several researchers have reported on the combination of psychotherapy and medication to be more effective compared to each of these therapies alone (15). Overall, dysthymic patients have shown to respond to anti-depressants and sometimes to psychotherapy. Of course, most of the patients who have been successfully treated by medication have residual symptoms and disturbed psychological function, which leads to interpersonal and social problems (16). Teasdale (1997), using the metaphor "mind-in-place" which he had borrowed from Robert Ornestein, states that we have got several minds each of which may be active for a moment. If this is not the case, mind-in-place occurs which means that one of the minds has been placed in the active status. In mood disorders, the individuals stick to one of these minds and the interaction between cognition and emotion plays a major role in the continuation of the minds. Teasdale (1999) believes that similar to stress disorders, emotional processing must be carried out for an effective treatment of depression. Hunt's study (1998) also showed emotional processing as a successful method for improving the effect of depressing events. Furthermore , Teasdale trained depressed patients using Mindfulness-Based Cognitive Therapy (MBCT), in order to prevent the recurrence of depression and direct the patients towards achieving a meta-cognitive insight (17). MBCT is a psychological treatment which combines Beck's cognitive therapy dimensions and Kabat-Zinn's mindfulness-based stress reduction program. It is identified as a treatment which works by paying attention to the present time and having a non-judgmental awareness of internal as well as external experiences (18). One of the basic assumptions of MBCT is that mind processes the experiences in two ways. The first way is the "functional" method which attempts to decrease the distance between the present situation and the desirable one by continuous problem solving. On the other hand, in the "being" or "understanding" method attempts are made in order to face the situations the way they are without trying to change them. The first method can be utilized in many dimensions of life; however, it is usually considered as inefficient in the view of depressed individuals. Thus, mindfulness aims to change the way of thinking towards the "being" method. In fact, this treatment method aims to teach the patients to deal with their thoughts and experiences in a different manner (19). In this way, while the patients acquire the mindfulness skills, they learn to accept less power and authority while they stop self judgment and blame thus factors which are in fact the nourishment of negative thoughts are eliminated. (20). Overall, MBCT teaches the patients that thoughts are only "thoughts" rather than "realities". This method enables the individuals to bring themselves back to the present in case they encounter primary symptoms of depression such as negative moods, negative thoughts, and inefficient processing methods through focusing on their breathing, physical emotions and their actions; thus, avoid being trapped by the "downward spiral" of depression (18). Overall, although MBCT protocol mainly aims to prevent the recurrence of depression symptoms in the temporary recovery phase, it can be an efficient treatment method in the active phase, even in chronic and treatment-resistant depression (21-23).

## 2. Objectives

This study aimed to evaluate the effectiveness of mindfulness-based cognitive therapy on a chronic type of depression disorder called dysthymia

## 3. Patients and Methods

### 3.1. Study Design

The present study was performed in two-group quasi-experimental or static comparison design in order to compare the effect of MBCT accompanied by pharmacotherapy and pharmacotherapy alone in treatment of dysthymia.

### 3.2. Study Subjects

Among the patients who had referred to two of the psychiatric clinics affiliated to Shiraz University of Medical Sciences, Shiraz, Iran, 75 were selected through convenience sampling. 50 individuals were enrolled in the study and randomly assigned to either the control or the case group. The control group received medication, while the experimental group received MBCT in addition to medication. The study groups completed the pre-test and the post-test questionnaires both before and after the intervention. All the participants filled out an informed consent form before entering the study. The inclusion criteria of the study were, suffering from depression for at least 2 years based on the diagnostic criteria of dysthymia, being above 18, having at least a diploma, not receiving any other psychological treatment elsewhere for the case group and not undergoing any psychological treatment while receiving medication for the control group, not being diagnosed with psychosis, mania, hypomania or personality disorders in the clinical interview, lack of drug or alcohol abuse while taking part in the study, not using medication for other diseases which might interfere with the treatment processes and not suffering from depression resulting from a simultaneous physical problem. Moreover, the subjects had to participate in at least 5 out of the 8 sessions in order to take part in the study.

### 3.3. Study Instruments

Beck Depression Inventory-2 (BDI-II): This questionnaire was used in order to evaluate the severity of the subjects’ depression. BDI-II is the new version of a 21-item, self-report questionnaire for assessing the severity of depression in adults as well as adolescents above 13. The items of this questionnaire are scored from 0-3 ranging from lack of a special symptom to the highest degree of that symptom. Furthermore, Mohammadkhani and Dobson conducted a study and showed BDI-II as a reliable and valid instrument for the Iranian population whose results can be reliably used for statistical as well as psychometric analyses (24). Five Facets of Mindfulness Questionnaire-18 (FFMQ): This questionnaire, designed by Baer et al. (2006), was used in order to assess mindfulness in the study subjects. FFQM is a 39-item questionnaire which evaluates 5 separate components of mindfulness including; observation, description, acting with awareness, non-judgmental and non-reacting, through the 5-option Likert scale. This questionnaire was standardized by Kalantari (2011, Iran). Moreover, the validity of the questionnaire was determined using factor analysis of the main components with varimax rotation.Furthermore, 7 questions were omitted from the questionnaire due to the lack of load factor on the intended factor or lack of a sufficient threshold in the load factor and thereby, 32 questions remained in the questionnaire (25). Structured clinical interview for diagnosis of axis I disorders in DSM-IV-TR: This interview was designed by First et al. (2002) and is used for diagnosis of dysthymia and eliminating differential diagnosis. It should be noted that the interview is reliable and valid for diagnosis of psychological disorders.

### 3.4. Treatment Process

The guidelines of the psychotherapy program were arranged based on “MBCT for depression” by Segal et al. (2002) and consisted of 8 weekly sessions each lasting for almost 2.5 hours. In the first session a clinical interview was performed and the treatment method was explained to the participants. The treatment protocol performed is shown briefly in the Table 1 (18).

**Table 1.** Curriculum for Sessions

Sessions	Contents of Each Session
**Session 1**	Establish orientation of the class and set the rules, raisin exercise to train being in the present moment, body scan practice , breath focus exercise
**Session 2**	Body scan practice, thought and feeling exercise, pleasant event calendar, mindfulness of routine activity
**Session 3**	Seeing and hearing exercise, sitting meditation, 3-minute breathing space, mindful walking, unpleasant event calendar
**Session 4**	Seeing and hearing exercise, sitting meditation, defining the territory of depression: negative automatic thought, diagnosis criteria for depression
**Session 5**	Sitting meditation, breathing space, reading poems related to mindfulness, introducing the concept of “Acceptance”
**Session 6**	Sitting meditation, mood, thoughts and alternative points exercise, breathing space, observing thoughts and feelings technique
**Session 7**	Sitting meditation, exercise to explore links between activity and mood, behavioral activation (generate a list of pleasure and mastery activities), identifying actions to do in low mood periods.
**Session 8**	Body scan practice, review the whole course, discuss how best to keep up what has been developed over the past 7weeks, discuss plans and positive reasons for maintaining the practice

## 4. Results

Three individuals were excluded from the study because of non-compliance of their work schedule, dissatisfaction of their family and the long distance between the location of classes (Hafiz hospital) and theirhome. In addition, 3 participants of the control group were not available for completing the post-test questionnaire. Therefore, the statistical analyses were performed on 44 participants in two 22-subject groups. Since the two study groups were analyzed independently and in order to control the pre-test's effect, ANCOVA was utilized in the present study. Of course, before conducting ANCOVA, the variables were investigated to ensure the necessary assumptions have been in place. About “depression” variable since the slopes of the regression lines were parallel and the data benefited from normal distribution, ANCOVA could be used. Table 2 shows the mean of the studied variables in the two study groups both before and after the intervention. As the table depicts, the case group's mean score of depression decreased from 31.3 before the intervention to 17 after the intervention. On the other hand, the control group's mean score of depression reduced from 27.5 before the intervention to 24.2 after the intervention. Considering mindfulness variables, the case group's mean score increased from 85.6 in the pre-test to 100.5 in the post-test. In addition, the control group's mean score was 95.8 and 97 in the pre-test and post-test, respectively.

**Table 2.** Comparison of the Two Groups' Mean Scores of Depression and Mindfulness Before and After the Intervention

Variable, Mean ± SD	Case		Control	
	Before the Treatment	After the Treatment	Before the Treatment	After the Treatment
**Depression**	31.3 ± 10.1	17 ± 12.2	27.5 ± 9.6	24.2 ± 8.7
**Mindfulness**	85.6 ± 10.7	100.5 ± 14.7	95.8 ± 11.4	97 ± 11.1

According to the two groups' mean and SD, the case group's depression scores have considerably increased in comparison to those of the control group and the results of ANCOVA showed the difference to be statistically significant (P = 0.000) (Table 3).

**Table 3.** The results of ANCOVA on thetwo groups

Source of Change	Sum of Squares	Degree of Freedom	Mean Squares	F Coefficient	P value
**Depression**	1306.4	1	1306.4	21.9	0.000
**Error**	2439.276	41	59.495	-	-
**Total**	24121	41	-	-	-

Regarding mindfulness, the results of linear regression analysis revealed that the slopes of the regression lines were not parallel in any of the two studied groups and the two groups' data did not follow a normal distribution; therefore, independent t-test was used in order to analyze the data obtained from these scales as well as their subscales. In doing so, the difference between the mean scores obtained from the subscales as well as the total scale in the pre-test and the post-test was computed and independent t-test was performed on the mean differences. The results are presented in Table 4.

**Table 4.** The results of independent T-test on the mean difference between the pre-test and the post-test

Source of Change	T-ratio	Mean Differences	P value
**Subscales**			
Observation	1.4	1.5	0.16
Description	0.2	0.45	0.8
Acting with awareness	2.8	5.8	0.007
Non-judgmental	4.1	5.3	0.000
Non-reacting	1	1.6	0.28
**Mindfulness**	3.36	15	0.002

## 5. Discussion

The first objective of the present study targeted the effectiveness of MBCT in dysthymia. One of the major purposes of this study was to determine the two groups' depression scores before and after the intervention. As Table 2 shows, the experimental group's depression mean score was 31.3 before the intervention and after the intervention, the experimental group's depression mean score decreased to 17. Thus, MBCT decreased the experimental group's depression mean score from severe level to mild level. As indicated by Table 3 the difference between the two groups is statistically significant which shows considerable reduction in depression of the experimental group compared to the control group. A large number of studies have shown the effectiveness of this method in preventing the recurrence and relapse of major depression (21, 26-30). Also, the effectiveness of MBCT in treatment-resistant depression and treating Major depression Disorder has been shown (23, 31) yet, only a limited number of studies have investigated the effect of MBCT on dysthymic patients. For instance, we can refer to Abolghasemi et al. (2008) (32), Hamidpour (2007) and Hamidpour and Sahebis’ (2005) findings which were in line with the results of the present study in treating dysthymia (33, 34). Regarding mindfulness, the results of Table 2 showed a significant increase in the case group's score compared to the control group. This may imply that performing MBCT has led to an increase in the components assessing mindfulness among the case group subjects. In addition, considering P = 0.00 and t = 3.36, it can be concluded that MBCT has significantly increased mindfulness among the participants of the case group in comparison to the control group. Table 4 also presents the results of the analysis of mindfulness subscales. Among the 5 subscales of observation, description, acting with awareness, non-judgmental and non-reacting, only acting with awareness and non-judgmental were significantly different between the two study groups. Description, which was not significantly different between the two groups, evaluates the individuals' ability for verbalizing their thoughts and emotions. Although the study subjects were advised to name their thoughts and emotions after identifying them, this component does not seem to have been influenced much by the interventions. This might be due to the fact that since the participants were trained to treat their thoughts and emotions the way they are and face them phenomenological, this has affected the individuals' verbalization of their experiences. The study results revealed a significant difference between the two groups regarding acting with awareness, which might be due to mindfulness course's emphasis on more conscious way of life and performance of tasks. Since acting with awareness is one of the most important subscales of mindfulness and the major issue of the present study, it has been assumed that awareness provides the ground for controlling as well as management of both mental and emotional conditions. The significant increase in the case group's score of non-judgmental factor also shows that one of the components of mindfulness which has caused considerable changes in the severity of depression among the experimental group is forgetting about judging and blaming oneself for what happened in the past and making attempts for accepting what is there at the present moment. This finding is in line with parts of the study by Kuyken et al. (2010) which considers self-compassion as well as mindfulness as the inducers of an affective MBCT. In fact, self-compassion overlaps with the concept of non-judgmental to a great extent. Nevertheless, other findings of the present study are not consistent with those observed by Kuyken et al. (2010) (20). In that study, the reduction in the severity of depression was attributed to non-reacting as well asself-compassion, while this component was not greatly affected in the present study. This difference might result from the fact that since the subjects of the current study were dysthymic patients who had suffered from depression for at least 2 years (some more than 10 years), the longtime spent dealing with symptoms, their thinking style and depressed mood had made depression a sustainable part of these people's lives. Moreover, although the 8-week treatment period seems to be appropriate for receiving, internalizing and theoretically accepting the concept of non-judgmental, its implementation in one's thoughts, feelings and life which is similar to non-reacting, requires a deeper change in the individuals' attitudes and insights. In the present study, the most significant changes in depression scores were related to the two basic features of MBCT; acting with awareness and non-judgmental. In fact, the increase in acting with awareness prevents the patients from being trapped by mental rumination and downward spiral and consequently, provides them with more control on their moods and emotions as well as awareness regarding their conditions. On the other hand, acceptance of one self and non-judgmental behavior neutralizes the toxic effects of blaming and comparing mental content of dysthymic patients. It means that there might be judging and blaming by the content of thoughts but they have least effect on the patients. One of the main limitations of this study was a non-random selection and small sample size of case and control groups that lead us to generalize these findings cautiously. Furthermore, lack of follow-up period due to time constraints was one the other limitations that suggest further researches to be considered to evaluate the maintenance of gains over time.
